# Exploring the relation of active surveillance schedules and prostate cancer mortality

**DOI:** 10.1002/cam4.6977

**Published:** 2024-03-16

**Authors:** Zhenwei Yang, Eveline A. M. Heijnsdijk, Lisa F. Newcomb, Dimitris Rizopoulos, Nicole S. Erler

**Affiliations:** ^1^ Department of Biostatistics Erasmus University Medical Center Rotterdam the Netherlands; ^2^ Department of Epidemiology Erasmus University Medical Center Rotterdam the Netherlands; ^3^ Department of Public Health Erasmus University Medical Center Rotterdam the Netherlands; ^4^ Cancer Prevention Program, Public Health Sciences, Fred Hutchinson Cancer Center Seattle Washington USA

**Keywords:** active surveillance, biopsy schedules, cancer mortality, detection delay, microsimulation model, prostate cancer

## Abstract

**Background:**

Active surveillance (AS), where treatment is deferred until cancer progression is detected by a biopsy, is acknowledged as a way to reduce overtreatment in prostate cancer. However, a consensus on the frequency of taking biopsies while in AS is lacking. In former studies to optimize biopsy schedules, the delay in progression detection was taken as an evaluation indicator and believed to be associated with the long‐term outcome, prostate cancer mortality. Nevertheless, this relation was never investigated in empirical data. Here, we use simulated data from a microsimulation model to fill this knowledge gap.

**Methods:**

In this study, the established MIcrosimulation SCreening Analysis model was extended with functionality to simulate the AS procedures. The biopsy sensitivity in the model was calibrated on the Canary Prostate Cancer Active Surveillance Study (PASS) data, and four (tri‐yearly, bi‐yearly, PASS, and yearly) AS programs were simulated. The relation between detection delay and prostate cancer mortality was investigated by Cox models.

**Results:**

The biopsy sensitivity of progression detection was found to be 50%. The Cox models show a positive relation between a longer detection delay and a higher risk of prostate cancer death. A 2‐year delay resulted in a prostate cancer death risk of 2.46%–2.69% 5 years after progression detection and a 10‐year risk of 5.75%–5.91%. A 4‐year delay led to an approximately 8% greater 5‐year risk and an approximately 25% greater 10‐year risk.

**Conclusion:**

The detection delay is confirmed as a surrogate for prostate cancer mortality. A cut‐off for a “safe” detection delay could not be identified.

## 1 INTRODUCTION

With the prostate‐specific antigen (PSA) test starting to play a role in screening for prostate cancer (PC), the European Randomized Study of Screening for Prostate Cancer (ERSPC) trial showed that PSA screening led to a 20% reduction in PC mortality.^1^ However, the accompanying and considerably increasing trends of diagnosis and treatment in PC raised concerns among researchers and policymakers.^2^, ^3^ Around 67% of patients with low‐risk PC are overtreated^4^ and likely to suffer from the adverse effects of invasive treatments, such as urinary incontinence and impotence.^5^

Due to the fact that most low‐risk diagnosed PC s are indolent, active surveillance (AS) with the idea of postponing the treatment for early‐stage PCs came into being.^6^ During AS, biopsies are taken to monitor the disease and detect progression. However, current AS cohorts employ different biopsy intervals since there is no consensus so far.^7^ For instance, the Johns Hopkins cohort requires annual biopsies for the participants,^8^ and the Prostate Cancer Active Surveillance Study (PASS) protocol calls for annual biopsies in the first 2 years and afterwards biennially.^9^ More frequent biopsies can provide timely detection of cancer progression at the cost of pain and potential infections. On the contrary, fewer biopsies can relieve patients' burden but are more likely to delay the detection and potentially miss the best window of opportunity for treatment. The main difficulty in choosing the optimal intervals for biopsies in AS lies in the limited knowledge about how much the delay in detecting cancer progression is and how this delay is related to PC mortality.

This study aims to evaluate the long‐term clinical outcome of delay in the detection of cancer progression in AS of PC. For this purpose, we extend the MIcrosimulation SCreening Analysis‐PROstate (MISCAN‐PRO) model to incorporate AS schedules and calibrate it on the Canary PASS data. The resulting detection delay and PC mortality are investigated using Cox proportional hazard models.

## 2 METHODS

### 2.1 Patient characteristics

The Canary PASS study is a multi‐center study in the US and Canada for managing PC patients with AS. Eligible patients are supposed to undergo regular prostate PSA tests (every 3 months), and prostate biopsies every year in the first 2 years and afterwards biennially.^9^ Eight hundred fifty patients with clinically localized PC and a Gleason score of six on both diagnostic and confirmatory biopsy were enrolled in the study between 2008 and 2017.

The calibration of the biopsy sensitivity to detect cancer progression was done using the subset of the 833 patients with at least one PSA measurement available for consistency with our previous research.^10^ The median follow‐up time calculated in the cohort, based on the reverse Kaplan–Meier,^11^ was 5.43 years (interquartile range: 3.49). A detailed summary of the data is presented in Table 1. Among the included patients, 183 patients were detected with progression, whereas 87 patients initiated treatment early, i.e., before progression detection. Thus, early treatment constitutes a competing event for the primary endpoint, cancer progression.

**TABLE 1:** Summary table for the Canary PASS data.

Item	Value
Number of patients	833
Follow‐up time (years)^a^	5.43 (3.76–7.25)
Baseline PSA density^c^ (ng/mL^2^)^b^	0.12 (0.10)
Age at start of AS (years)^a^	62 (57–67)
Total number of PSA measurements	8262
Number of PSA measurements per patient^a^	9 (5–14)
PSA level (ng/mL)^b^	5.10 (3.84)
Number of positive cores per patient^a^	3 (2–4)
Percentage of positive cores (%)^a^	8.33 (0.0–16.67)
Number of biopsies per patient^a^	2 (2–3)

Abbreviations: AS, active surveillance; PASS, Prostate Cancer Active Surveillance Study; PSA, prostate‐specific antigen.

^a^ Median is shown followed by the interval between 25% quantile and 75% quantile.

^b^ Mean is shown with standard deviation in the brackets.

^c^ PSA density equals to PSA level (ng/mL) divided by prostate volume (mL).

### 2.2 MISCAN model

The MISCAN‐PRO model is a microsimulation model that simulates individuals' PC‐related life trajectories by imposing pre‐determined probabilities of tumor onset and tumor growth (described in detail at http://cisnet.cancer.gov/prostate/profiles.html).^12^ The natural history of PC is captured by 18 cancer states, namely the combination of three stages (T1, T2, and T3), Gleason score (<7, 7, >7), and metastasis (M0, M1), and men move through these states according to specific transition probabilities. In addition, we distinguish the pre‐clinical stage, when no symptoms have shown, and the clinical stage, after symptoms have developed. If a patient is diagnosed with PC, he is referred to one of three management options, radiotherapy (RT), radical prostatectomy (RP), or AS, and the time of PC‐related death is simulated depending on the patient's current age and tumor state. This calculation is based on Surveillance, Epidemiology, and End Results (SEER) data from the pre‐PSA era 1983–1986 for the cases detected clinically and corrected with a factor taking into account survival has improved since then.^13^ In addition, better survival is assumed for non‐metastatic cases who receive RT or RP, using a hazard ratio of 0.56.

To model disease progression after entering AS, we extended this original MISCAN‐PRO model. In this extension, all low‐risk patients, defined as those with early stage (i.e., <T3), Gleason score <7, and no metastases (M0), are assigned to AS. During AS, patients continue their trajectory through the cancer states, and biopsies are scheduled according to a pre‐specified schedule and subject to a certain probability of compliance. Biopsies detect cancer progression, defined as a Gleason score ≥7, stage T3, or metastasis (M1), with a certain probability (biopsy sensitivity, which needs to be calibrated). An active treatment (RT or RP) is given when progression is detected, and AS ends. Patients diagnosed by screening but do not yet have clinical symptoms are assumed to have a lead time and can be cured from PC with a probability depending on this lead time. The lead time is defined as the duration between progression detection and the simulated time of symptom onset if there were no interventions (e.g., screening or AS). For patients who are not cured and patients who already show symptoms at the start of AS, the time of PC death is updated conditional on the age and state at progression detection. Every patient is initially simulated with a death time for other causes, based on the life table in the Netherlands. Patients whose PC death is ahead of this time point will die from PC.

All parameters related to the natural history (i.e., onset probabilities, duration on each state, transition probabilities, etc.) were calibrated on the ERSPC data.^13^ Since the model simulates the underlying disease, some quantities that are unobserved in real‐world data, like the time of cancer progression (depicted in detail in Section 2.5), can be directly derived from the simulation.

### 2.3 Calibration of the biopsy sensitivity

The sensitivity of the biopsies to detect PC progression was calibrated to match the cumulative incidence of progression detection estimated from the Canary PASS data. We simulated four identical populations of 1 million men and assumed different biopsy sensitivities (40%, 50%, 60%, 70%). The inclusion criteria, a Gleason score of six, T1/T2, and no metastasis, and the assumed compliance rate of 65%, matched those of the Canary PASS trial. For the calibration, cancer progression was defined as only Gleason score upgrading to seven or more. Considering that in the Canary PASS trial, patients seeking early treatment is a competing risk to cancer progression, the observed cumulative incidence of cancer progression was derived based on the Aalen‐Johansen estimator.^14^ In the simulated data, where there was only uninformative censoring, the Kaplan–Meier estimator was used.

### 2.4 Simulation of pseudo study groups (biopsy schedules)

Using the calibrated model, we compared four scenarios of fixed biopsy schedules in the same simulated population. In each scenario, 10 million people born in 1970 and followed until death were simulated. A biennial screening program for PC between the age 55 and 69 was implemented, and patients who were (either screening or clinically) diagnosed with localized PC (Gleason score of six, T1/T2, and no metastasis) and aged ≥55 years old were eligible for AS. In all scenarios, we assumed the calibrated sensitivity (50%), a compliance rate of 100%, and a random dropout rate of 10%. The scenarios differed in the AS biopsy schedules, namely a tri‐yearly schedule (biopsies every 3 years), bi‐yearly schedule (biopsies every other year), PASS schedule (biennial biopsies after the first year), and a yearly schedule (biopsies every year). In the following analysis, only the simulated patients who were detected with cancer progression were included in the study group of each scenario.

### 2.5 Outcomes

In the context of the simulated pseudo study groups under four AS schedules, the individual data are of the most interest. Utilizing the data, we will focus on the primary outcomes and secondary outcomes as follows.

#### 2.5.1 Primary outcomes

For each individual, their underlying disease development trajectory and the survival outcome, mortality, were directly derived from the MISCAN model. With the time of underlying progression and the pre‐specified age when biopsies indicated cancer progression (i.e., progression detection), the detection delay was calculated. Section 2.6 explains how the relationship between PC death detection delay was investigated while considering other covariates.

#### 2.5.2 Secondary outcomes

The secondary aim is to compare the effectiveness and efficiency of the four AS schedules. Therefore, five quantities are reported: overtreatment, life years gained per 1000 patients, life years gained per averted PC death, mean detection delay, and the 10‐year PC mortality. The latter two quantities are straightforwardly extracted from the simulated data. The concept and calculation of the former three quantities can be found in the Data S1.

### 2.6 Statistical analysis

**FIGURE 1:**
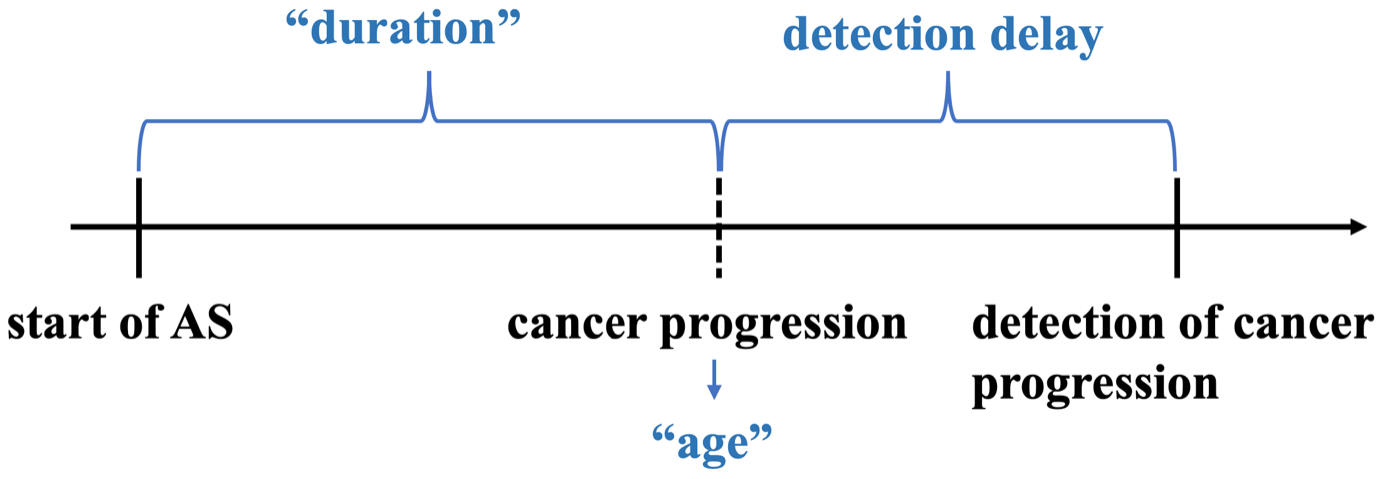
The illustration of five concepts, start of active surveillance (AS), cancer progression (i.e., age at cancer progression), detection of cancer progression, duration (time on AS until cancer progression), and detection delay.

For primary analysis, cox models were fitted on the data resulting from each of the four simulated scenarios to investigate the relation between detection delay and PC mortality. The model formula is

$$\begin{array}{c} h_{i}\left(t\right)=h_{0}\left(t\right)\exp\{{\text{duration}}_{i}+{\mathrm{age}}_{i}+C\left({\text{delay}}_{i},3\right)\\ +C\left({\text{delay}}_{i},3\right)\times {\mathrm{age}}_{i}+C\left({\text{delay}}_{i},3\right)\times {\text{duration}}_{i}, \end{array}$$

where, $h_{i}\left(t\right)$ is the hazard of prostate cancer death at time t for patient i, $h_{0}\left(t\right)$ denotes the baseline hazard at time t. The time from the start of AS until cancer progression is denoted as “duration”, “age” stands for the age at time of cancer progression, and “delay” stands for the detection delay (time difference between cancer progression and detection of cancer progression). The concepts of these three covariates are illustrated in Figure 1. The time scale t starts from the detection of cancer progression and thus does not overlap with the time periods “duration” and “delay”. The shape of the effect of detection delay on PC mortality was investigated using natural cubic splines (with three degrees of freedom), $C\left({\text{delay}}_{i},3\right)$, and was allowed to differ across patients' age and the AS “duration” by including interaction terms. The models were fitted in R 4.2.2.^15^ To facilitate the interpretation of the association between detection delay and PC mortality, we provide effect plots showing the expected log hazard for six combinations of duration on AS (2, 4, 8 years) and age at progression (66, 72 years old). In addition, the risks of PC death were calculated from the Cox models and visualized for the median duration (3.89 years) and age (69 years old).

## 3 RESULTS

### 3.1 Calibration of the biopsy sensitivity

**FIGURE 2:**
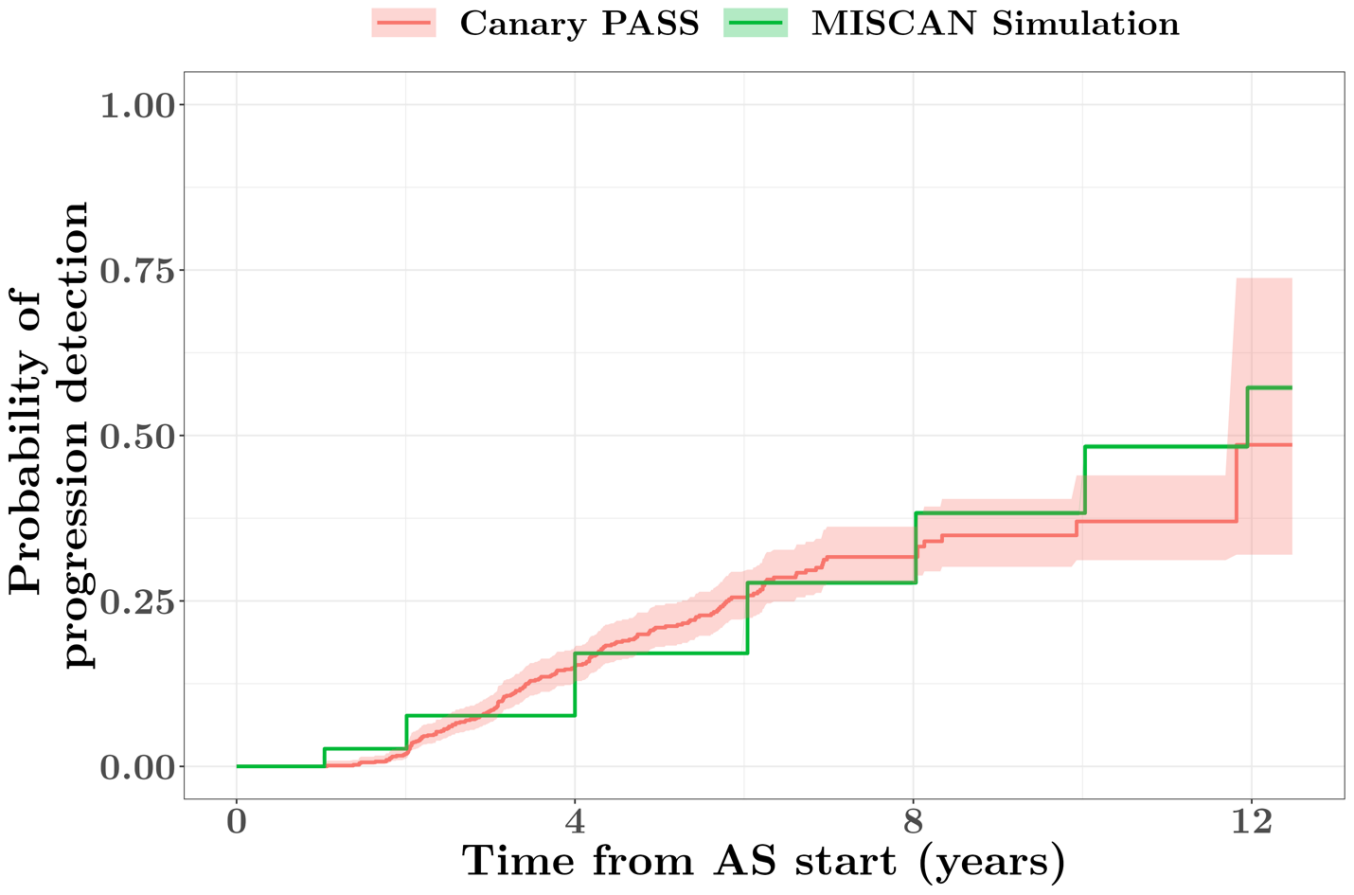
The cumulative incidence of detected progression in the simulated data (green line; Kaplan–Meier estimate) and the Canary Prostate Cancer Active Surveillance Study (PASS) data (red line; Aalen‐Johansen estimate), assuming a biopsy sensitivity of 50%. AS, active surveillance; MISCAN, MIcrosimulation SCreening Analysis.

The cumulative incidences of progression detection in the simulated data were compared to the cumulative incidence estimated in the Canary PASS data. The assumption of a biopsy sensitivity of 50% resulted in a cumulative incidence of cancer progression similar to that estimated in the Canary PASS data (Figure 2).

### 3.2 Primary outcomes: Impact of detection delay on PC mortality

**FIGURE 3:**
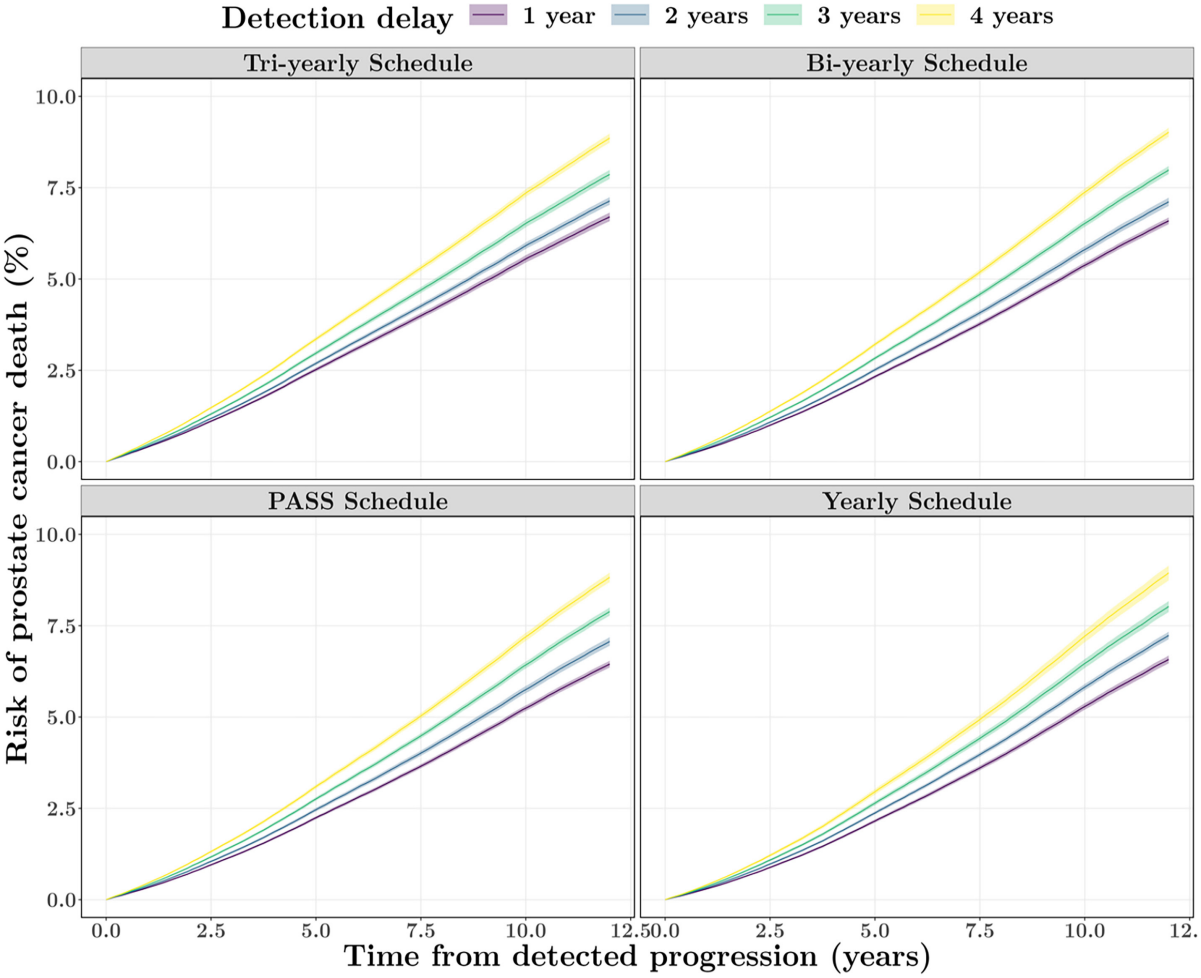
The predicted cumulative risks of prostate cancer death for a detection delay of 1–4 years across four fixed biopsy schedule scenarios, conditional on a progression happening 3.89 years after the start of active surveillance (AS) and patients being 69 years old at the time of progression. PASS, Prostate Cancer Active Surveillance Study.

To visualize the relation between the delay in detection and PC mortality across the four biopsy schedules, we present the estimated cumulative risk of PC death 12 years after the detection of progression in Figure 3. The 5‐ and 10‐year risk estimates with 95% confidence intervals are summarized in Table 2. In all scenarios, a longer detection delay increased the risk of PC death. The difference in the cumulative risk between a 3‐year and 2‐year delay or a 4‐year and 3‐year delay was more extensive than that between a 2‐year and 1‐year delay. More specifically, compared with a 2‐year delay, a 4‐year delay led to a ~8% greater 5‐year risk and a ~25% increase in the 10‐year risk. Schedules with higher biopsy frequency had a significantly lower PC mortality (by ∼8% when comparing the tri‐yearly and yearly schedule and by ∼4% when comparing the bi‐yearly and yearly schedule). The 5‐year risk in the PASS and bi‐yearly schedule were (almost) the same for delays up to 4 years. The 10‐year risk in the PASS schedule was lower than that in the yearly schedule, but there was no evidence of a difference in this case.

**TABLE 2:** The 5‐year and 10‐year risks of PC death conditional on a delay of 1–10 years across four biopsy schedules.

Delay	Schedule			
	Tri‐yearly	Bi‐yearly	PASS	Yearly
5‐year risks (%) [95% CI]				
1‐year	2.52 [2.47, 2.57]	2.33 [2.28, 2.37]	2.42 [2.20, 2.29]	2.15 [2.11, 2.20]
2‐year	2.69 [2.64, 2.74]	2.51 [2.46, 2.57]	2.46 [2.41, 2.52]	2.37 [2.33, 2.42]
3‐year	2.97 [2.91, 3.03]	2.83 [2.78, 2.88]	2.76 [2.70, 2.81]	2.64 [2.58, 2.70]
4‐year	3.36 [3.30, 3.42]	3.21 [3.15, 3.27]	3.09 [3.04, 3.15]	2.95 [2.87, 3.03]
10‐year risks (%) [95% CI]				
1‐year	5.55 [5.45, 5.65]	5.38 [5.29, 5.46]	5.24 [5.16, 5.32]	5.29 [5.20, 5.38]
2‐year	5.91 [5.82, 6.00]	5.80 [5.71, 5.90]	5.75 [5.65, 5.85]	5.82 [5.73, 5.91]
3‐year	6.52 [6.41, 6.63]	6.52 [6.42, 6.61]	6.42 [6.33, 6.52]	6.46 [6.34, 6.59]
4‐year	7.35 [7.24, 7.46]	7.37 [7.27, 7.48]	7.19 [7.08, 7.30]	7.21 [7.04, 7.38]

Abbreviations: CI, confidence interval; PASS, Prostate Cancer Active Surveillance Study.

**FIGURE 4:**
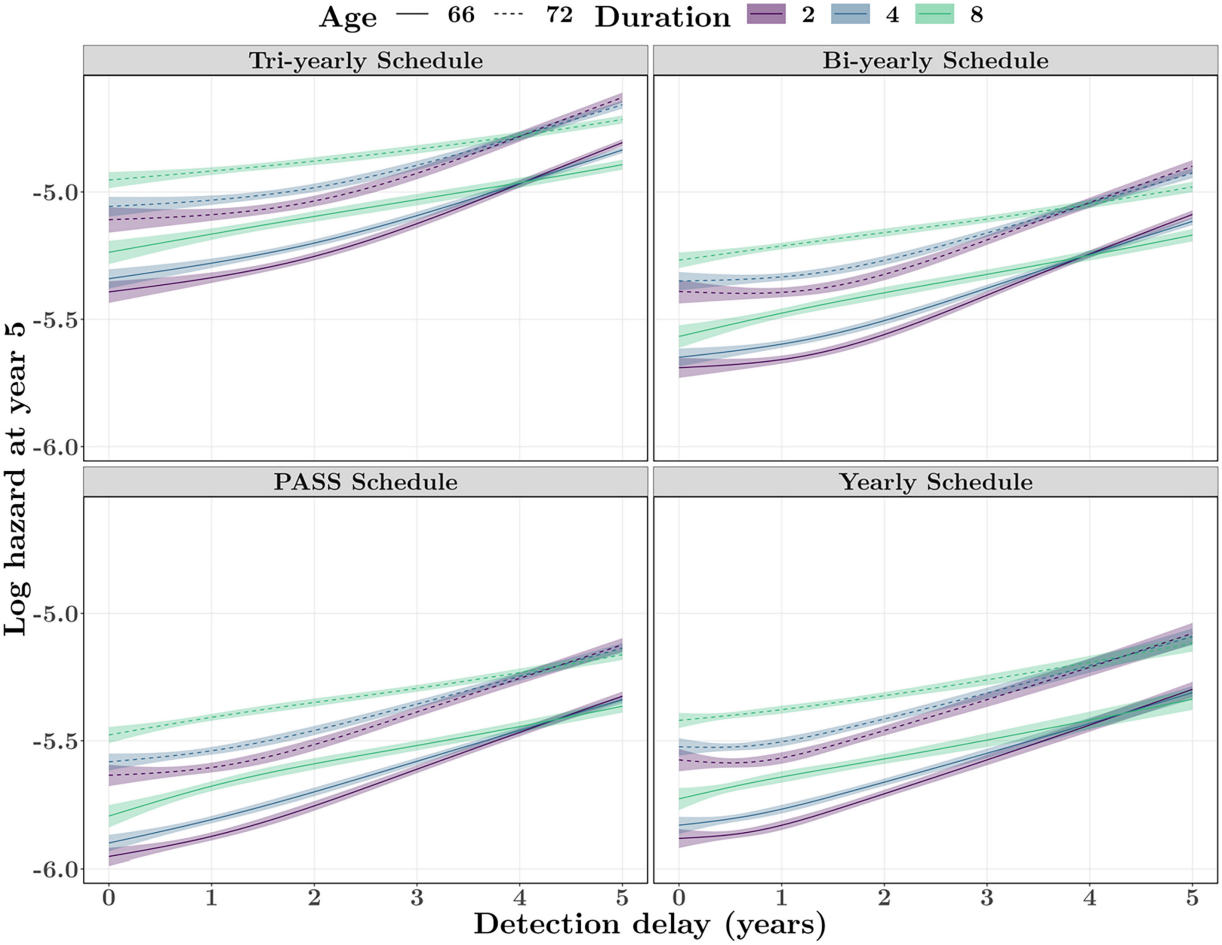
Effect plots for the impact of detection delay (i.e., time between cancer progression and detection of cancer progression) on prostate cancer mortality for four fixed biopsy schedule scenarios (duration: time from active surveillance [AS] start until cancer progression; age: age at cancer progression). PASS, Prostate Cancer Active Surveillance Study.

Furthermore, the impact of the detection delay on PC mortality was expected to be non‐linear and to interact with age and duration, as displayed in effect plots in Figure 4. They show the log hazard of PC mortality across detection delay for different ages and durations on AS. The PC mortality hazard in the tri‐yearly schedule was, on average, higher than those in all the other scenarios, while the hazards in the yearly and PASS schedules were comparable. Overall, an increasing detection delay was generally associated with increased PC mortality regardless of the biopsy frequency. This increase was smaller with a short delay (i.e., 1.5 years) in less frequent biopsy schedules, e.g., the tri‐yearly and bi‐yearly schedules. In addition, older age at the time of cancer progression was associated with higher PC mortality. The effect of duration was more complicated. With a shorter detection delay (<4 years), the patients who stayed longer in AS before underlying progression were more likely to die from PC, whereas the faster‐progressing patients who stayed a shorter period in AS and had a longer detection delay had a higher risk of PC death.

### 3.3 Secondary outcomes: Comparison of different biopsy schedules

From the whole simulated cohort (*N* = 10,000,000), 686,843 patients entered the AS programs. Estimated secondary outcomes according to each biopsy schedule were reported (Table 3). Specifically, the least frequent schedule, the tri‐yearly biopsy schedule, detected the fewest (530341) cases of cancer progression. However, this schedule was the most efficient in reducing overtreatment and was able to save, on average, 8.23 years of life per patient on AS. In contrast, more frequent schedules were more powerful in detecting cancer progression but at the cost of higher overtreatment, tending to waste 0.93 (13%), 1.08 (15%), and 1.98 (32%) more years under unnecessary treatment per patient in the bi‐yearly, PASS and yearly schedules than in the tri‐yearly schedules, respectively. Compared to the tri‐yearly schedule, the bi‐yearly, PASS, and yearly schedules gained 35.48, 59.43, and 63.07 life years per 1000 patients. Taking into account PC mortality, the PASS schedule outperformed the other two and gained 7.0 life years per avoided death compared to the tri‐yearly schedule. The 10‐year PC mortality was approximately 1.7% in all four scenarios.

**TABLE 3:** Clinical outcomes for four simulated biopsy schedules.

Quantity	Schedule			
	Tri‐yearly	Bi‐yearly	PASS	Yearly
Number of patients on AS	686,843	686,843	686,843	686,843
Number of progressed patients	530,341	558,266	560,286	583,791
Proportion of overtreated patients (%)	91.62	92.23	92.47	92.79
Overtreatment (years)	8.23	7.30	7.15	6.25
Life years gained per 1000 patients	‐	35.48	59.43	63.07
Life years gained per averted PC death	‐	5.74	6.98	5.39
Mean detection delay (years)	3.94	2.78	2.57	1.45
10‐year PC‐specific mortality (%)	1.73	1.68	1.65	1.69

Abbreviations: AS, active surveillance; PASS, Prostate Cancer Active Surveillance Study; PC, prostate cancer.

## 4 DISCUSSION

AS has been recently recommended as a management procedure for low‐risk PC in the PC guidelines from the American Society of Clinical Oncology,^16^ the European Association of Urology,^17^ etc. However, there is no clear statement about the intervals of biopsies conducted during AS, and most existing AS cohorts used different schedules, for biopsies, PSA measurements and so on. The concept of delay in cancer detection has been proposed and approximately estimated previously in determining the clinical outcomes of different screening intervals,^18^ whereas the counterpart in AS, delay in detection of cancer progression, is seldom discussed. Delay in detection of cancer progression, is difficult to approximate using real world data since the time of cancer progression is unknown. However, when using simulation models, we are able to construct the quantity of detection delay. On the other hand, a real‐world cohort can hardly follow the patients for more than 15 years due to the issue of expense. Thus studies in similar areas do not usually focus on the long‐term outcomes such as mortality, let alone its relation with an unobserved quantity, detection delay. Nevertheless, the simulation models are exempted from this restriction. Our study is the first one performing a simulation study, according to the simulation model that is based on multiple large cohorts, to give a clinical insight of detection delay under different AS scenarios and its long‐term effect.

To disclose the relation between detection delay and PC mortality over different biopsy intervals, the first step was to extend the existing simulation model, the MISCAN‐PRO model with the functionality to emulate the practice procedure of AS, allowing users to mimic any pre‐specified biopsy schedule. We utilized the Canary PASS data to calibrate the sensitivity of biopsies to detect PC progression and found that a biopsy sensitivity of 50% resulted in a comparable cumulative incidence of progression detection. The model parameters of the MISCAN‐PRO model used to simulate the disease progress of the patients (i.e., except the biopsy sensitivity) were calibrated on the ERSPC data in which 6‐core biopsy schemes were used. The use of such lower‐sensitivity biopsies may have led to misclassification of men to Gleason 6, and thereby an overestimation of the risk of progression of men on AS. When calibrating the biopsy sensitivity in the MISCAN‐PRO model to the Canary PASS in which biopsies with 12 or more cores (and likely better sensitivity) were used, the exaggerated progression rate may have resulted in a calibrated biopsy sensitivity to detect progression that is lower than in other studies.^19^

With the calibrated biopsy sensitivity, four AS scenarios following different fixed biopsy schedules were simulated. Overall, more frequent biopsy schedules were found to gain more life years but at the cost of lower efficiency in reducing overtreatment. This is in line with the findings by De Carvalho et al.^20^ who compared a schedule of tri‐yearly biopsies after the first year with a yearly schedule in the AS of a screen‐detected population and found that fewer biopsies reduced the overtreatment by up to 30% while there was a small increase in PC mortality. In our study, the PASS schedule (biennial biopsies after 1 year) was comparable to other biopsy schedules with regards to PC mortality and superior in terms of gained life years. Similarly, a previous study focused on the estimated risk of cancer progression and found that the PASS schedule resulted in comparable risks and detection delays to other AS cohorts and annual biopsies.^21^ We also found that a longer detection delay was associated with a higher risk of PC death. However, there was no clear cut‐off for a detection delay to be considered safe. Moreover, an exploratory utility analysis investigating the relationship between the detection delay and the Quality Adjusted Life Years (using the utility functions proposed by Heijnsdijk et al.^22^) did not find any plateaus or other nonlinearities from which a threshold for an acceptable detection delay could be derived (results not shown).

One particular strength of our study is the use of the MISCAN‐PRO model. The MISCAN‐PRO model is a well‐established modeling framework and was calibrated on one of the largest cohorts with a longer follow‐up, the ERSPC data,^13^ and we calibrated the AS part to a large AS cohort, the PASS study. The MISCAN‐PRO model simulates the patients' history of PC based on T‐stage, Gleason score, and metastases, which aligns with the eligibility criteria of most AS cohorts. This facilitates mimicking the AS programs in practice and provides add‐on information to the empirical data where cancer progression is never exactly observed. Our extension of the MISCAN‐PRO model is beneficial in multiple aspects. We investigated the long‐term outcomes of different biopsy schedules, serving as a foundation of potential cost‐effectiveness analyses for policy‐making on a population level. On an individual level, the exploration of the relation between detection delay and PC mortality provides crucial insight into the personalized AS scheduling field,^10^, ^23^ where schedules are optimized between fewer biopsies and longer detection delay (used as a surrogate for PC mortality).

This research; however, is subject to some potential limitations. First, the Canary PASS data used to calibrate the sensitivity of a biopsy have generally short follow‐ups (a median of 5.43 years). In addition, the lack of data about the effect of treatment delay due to AS makes it challenging to model the impact of AS in microsimulation models. Specifically, the probability of treatment curing patients from PC was only calibrated for patients immediately receiving treatment (instead of AS), but not for patients under AS, who receive treatment only after progression is detected. The predicted 10‐year PC mortalities, around 1.7%, is slightly above the reported range of 0%–1% in AS cohorts with similar inclusion criteria.^24^ This may be attributed to patients with more severe disease quitting AS to seek active treatment while in the simulation we assumed perfect compliance. In the model, the underlying stage distributions and progression rates are calibrated to the ERSPC trial. Since in the trial often 6 core biopsies were taken, the misclassification of Gleason 7 cancers as Gleason 6 cancers might be higher in the ERSPC and therefore the progression rates might be overestimated. Moreover, since a delay in detection is only defined for patients who survive until progression detection, the analysis of the relation between detection delay and PC mortality includes fewer patients when less frequent biopsy schedules are used, likely introducing selection bias. However, the relation between detection delay and PC mortality was very similar in the four schedules, which aligns with what we expect in the absence of selection bias. A fairly recent development is the use of MRI in AS, either as an alternative to biopsies or as guidance to increase the accuracy of biopsies.^25^ The MISCAN‐PRO model could be further extended to include MRI as a monitoring modality once data from AS cohorts with sufficient sample size and follow‐up time becomes available.

This study helps to quantify the effect of detection delay on clinical outcomes and confirms the detection delay as a surrogate of PC mortality. This, on the one hand, facilitates empirical studies in which people evaluate an AS cohort under the detection delay calculated from the approximation of cancer progression to compensate for the lack of mortality information. On the other hand, the extended MISCAN‐PRO model, as an evidence‐based model that integrated several large cohort datasets over the world, can be utilized as a trustable tool to investigate cost‐effectiveness of different AS schedules, with detection delay as an additional factor in choosing the optimal biopsy frequencies in AS. Within this context, the simulation‐based research is beneficial in delivering evidence of the PC management for multiple stakeholders, such as clinicians and policy makers.

## AUTHOR CONTRIBUTIONS

**Zhenwei Yang:** Conceptualization (equal); data curation (equal); formal analysis (equal); investigation (equal); methodology (equal); validation (equal); visualization (equal); writing – original draft (equal); writing – review and editing (equal). **Eveline Heijnsdijk:** Conceptualization (equal); investigation (equal); methodology (equal); supervision (equal); writing – review and editing (equal). **Lisa F. Newcomb:** Funding acquisition (equal); resources (equal); writing – review and editing (equal). **Dimitris Rizopoulos:** Conceptualization (equal); investigation (equal); methodology (equal); supervision (equal); writing – review and editing (equal). **Nicole S. Erler:** Conceptualization (equal); investigation (equal); methodology (equal); supervision (equal); writing – review and editing (equal).

## CONFLICT OF INTEREST STATEMENT

The study sponsors had no role in the study design; collection, analysis, or interpretation of data; writing of the report; or the decision to submit the manuscript for publication. This work's contents are solely the responsibility of the authors and do not necessarily represent the official views of the NCI.

## ETHICS STATEMENT

Since this study used pre‐existing data for which approval had been obtained before data collection, the Medical Ethics Committee of the Erasmus Medical Center has determined that this study does not fall under the Medical Research Involving Human Subjects Act and no further approval was required.

## Supporting information


Data S1.

## Data Availability

The data used to calibrate the biopsy sensitivity in this study are not publicly available due to privacy restrictions. The data can be requested from the PASS Scientific Review Committee (https://canarypass.org/researchers/).
