# Supplementary material for: Exploring the relation of active surveillance schedules and prostate cancer mortality
Source: Cancer Med. 2024 Mar 16;13(5):e6977. doi: 10.1002/cam4.6977 (PMC10943374; doi:10.1002/cam4.6977)
Supplement: Supplementary file 1 — Data S1. [file CAM4-13-e6977-s001.docx]

**Exploring the Relation of Active Surveillance Schedules and Prostate Cancer Mortality**

Zhenwei Yang, MSc^1,2,*^, Eveline A.M. Heijnsdijk, PhD^3^, Lisa F. Newcomb, PhD^4^, Dimitris Rizopoulos, PhD^1,2^, and Nicole S. Erler, PhD^1,2^

^1^Department of Biostatistics, Erasmus University Medical Center, Rotterdam, the Netherlands

^2^Department of Epidemiology, Erasmus University Medical Center, Rotterdam, the Netherlands

^3^Department of Public Health, Erasmus University Medical Center, Rotterdam, the Netherlands

^4^Cancer Prevention Program, Public Health Sciences, Fred Hutchinson Cancer Center, Seattle, Washington, USA

*Corresponding author

Zhenwei Yang, MSc

Department of Biostatistics, Erasmus University Medical Center

Na-2818, Wytemaweg 80, 3015CN, Rotterdam, the Netherlands

Email: [z.yang@erasmusmc.nl](mailto:z.yang@erasmusmc.nl)

Phone (work): +31 10 70 43395

**Interpretation example of Table 2**

For one patient who undergoes a *tri-yearly schedule* (where biopsies are conducted every three years), his risk of dying from prostate cancer *five years after the detection of his cancer progression* is expected to be *2.52%* if he has one year of detection delay (i.e., his cancer progression was detected one year after it actually occurred). And this risk rises to *5.55%* *ten years after the detection of his cancer progression*.

**Concept and calculation of overtreatment**

Overtreatment stands for the unnecessary active treatment that has been imposed on the patients who could have lived their life without having to suffer from the downsides of the treatment at all or too early. As known, the population screening program is the biggest trigger of overtreatment whereas active surveillance (AS) is a way to reduce or delay it. In this study, we quantify the delay of overtreatment in the unit of time (years), which is how much time on unnecessary treatment on average per patient could be avoided by a specific AS program. The formula is：

Delay in $\text{overtreatment}= \frac{\text{treatment delay for patients dying from other causes}}{\text{number of AS patients}}$.

The quantity is calculated only among simulated patients who were recorded with death from other causes. Because even under AS (i.e., a delayed active treatment) they did not die from prostate cancer, we know that receiving an active treatment immediately would not have changed their outcome. The calculated value shows that the specific AS schedule is able to reduce the overtreatment by at least this amount.

**Concept and calculation of life years gained**

Both the life years gained per 1000 patients and life years gained per averted PC death are calculated relative to the tri-yearly schedule. With the least frequent biopsies, patients in the tri-yearly schedules have the highest mortality and shortest overall life expectancy. For example, the life years gained per 1000 patients for the bi-yearly schedule is calculated as:

$\frac{\text{difference in the sum of life expectancy in the bi-yearly and tri-yearly schedule}}{\text{number of patients under AS}}\times1000$.

The life years gained per averted PC death for the bi-yearly schedule is calculated as:

$$\frac{\text{difference in the sum of life expectancy in the bi-yearly and tri-yearly schedule}}{\text{difference in the number of patients dying from PC in the tri-yearly and bi-yearly schedule}}.$$
